# Perceptions of and willingness to participate in genomics research among a U.S. sample

**DOI:** 10.3389/fgene.2026.1808576

**Published:** 2026-09-09

**Authors:** Emerson Dusic, Devin Madden, Isabella E. Rios, Bo J. Hwang, Sima Rabinowitz, Michelle A. Ramos, David R. Pletta, Rodrigo A. Aguayo-Romero, Mitchell R. Lunn, Kellan Baker, Carol R. Horowitz, Sari L. Reisner

**Affiliations:** 1 Department of Epidemiology, University of Michigan School of Public Health, Ann Arbor, MI, United States; 2 Center for Social Epidemiology and Population Health, University of Michigan School of Public Health, Ann Arbor, MI, United States; 3 Institute for Health Equity Research, Icahn School of Medicine at Mount Sinai, New York, NY, United States; 4 Independent Researcher, Ann Arbor, MI, United States; 5 Institute for Health Research and Policy at Whitman-Walker, Washington, DC, United States; 6 The PRIDE Study/PRIDEnet, Stanford University School of Medicine, Stanford, CA, United States; 7 Department of Medicine, Division of Nephrology, Stanford University School of Medicine, Stanford, CA, United States; 8 Department of Epidemiology and Population Health, Stanford University School of Medicine, Stanford, CA, United States

**Keywords:** bioethics, genetics, genomics research, identity, transgender

## Abstract

**Introduction:**

The proliferation of biobanks and the collection of gender identity data have created opportunities to conduct transgender identity genetic research (TIGR), which explores possible genetic contributions to gender identity. However, without meaningfully engaging trans communities, researchers may struggle to understand and prevent potential harms while maximizing potential benefits, particularly when research fails to incorporate the social, medical, and political factors and environments shaping transgender peoples’ lives. This study explored factors associated with transgender individuals’ perspectives on and willingness to participate in TIGR.

**Methods:**

Between January and May 2025, we recruited a nationwide sample (n = 495) of transgender adults. Participants completed a cross-sectional survey assessing sociodemographics, policy safety, health, TIGR perspectives, and willingness to participate in TIGR. Regression models assessed correlates of TIGR perspectives and willingness to participate.

**Results:**

Participants’ median age was 29 years (IQR = 25–34); 38.4% were people of color. While 62.2% would be willing to participate, 68.9% agreed TIGR could harm transgender communities. More positive TIGR perspectives were observed among people of color (aβ = 0.48, 95% CI = 0.18 to 0.56) and those experiencing food insecurity (aβ = 0.21, 95% CI = 0.02 to 0.40). Less favorable perspectives were associated with disability (aβ = –0.20, 95% CI = –0.38 to –0.02), medical gender affirmation (aβ = –0.30, 95% CI = –0.54 to –0.06), mixed vs. negative transgender policy environment (aβ = –0.32, 95% CI = –0.59 to –0.05), and high medical mistrust (aβ = –0.24, 95% CI = –0.46 to –0.03). Positive perspectives were strongly associated with willingness to participate in TIGR (aOR = 14.48, 95% CI = 7.30 to 28.70).

**Discussion:**

Although most participants were willing to participate in TIGR, a substantial minority were unsure or unwilling, reflecting concerns about trust, risk, and benefits. Future genomics research involving transgender people must prioritize trust-building efforts, particularly those that ensure participant safety, to encourage future research participation.

## Introduction

Biological essentialism holds that identity and traits are intrinsic, objective, and purely biological (Ventresca et al., 2024; Sabatello and Juengst, 2019; Rajkovic et al., 2022; Lipsitz Bem, 1993). In genetics, essentialist thinking often manifests as genetic determinism—the assumption that genes directly and uniformly cause complex outcomes—obscuring polygenic architectures, gene–environment interplay, and social conditions that shape phenotypic expression (Sabatello and Juengst, 2019). When applied to socially constructed categories (*e.g.*, race, sexual orientation, gender, and IQ), this framing can be interpreted by both scientists and the general public as evidence of innate, fixed group differences, reinforcing harmful stereotypes and legitimizing discrimination. These interpretive risks are especially pronounced in behavioral genetics, a field that seeks to identify genetic correlates of complex, socially defined behaviors and traits whose measurement and meaning are inseparable from historical and cultural context (Charney, 2017). Misuse and misrepresentation of behavioral genetic research have contributed to many instances of real-world harms. For example, purported genetic explanations for racial inequities have contributed to increased stigma against Black communities and have motivated extremist acts of violence (Roberts, 2011), including the 2022 Buffalo grocery store mass shooting (Molteni, 2022). Essentialist narratives can also shape how research benefits and harms are distributed, raising questions about who sets research agendas, governs biobanks and data access, and defines acceptable downstream uses. This history underscores genomics researchers’ responsibility to reject narratives of inherent biological differences between social groups as misinterpreted findings can harm and erode trust, ultimately reducing marginalized communities’ willingness to participate in this research.

Transgender identity genomic research (TIGR) is a field of behavioral genetics that explores possible genetic determinants of, or contributions to, gender identity, dating back to the mid-twentieth century. Original TIGR studies took the form of case studies or series karyotyping transgender, nonbinary, and gender-diverse (hereafter, “trans”) clinical patients to identify potential variations in sex chromosomes but have mostly yielded insignificant findings (Levin et al., 2023; Fernández et al., 2014). Family and twin (both monozygotic and dizygotic) studies suggest some degree of heritability, although exact estimates vary (Levin et al., 2023; Coolidge et al., 2002; Polderman et al., 2018). Candidate gene studies, often guided by the Prenatal Androgen Hypothesis—which posits that variations in fetal androgen exposure influence gender identity—have examined genes related to hormone receptors (Fernández et al., 2015), but no candidate gene studies have been replicated (Levin et al., 2023). Whole exome sequencing studies suggest potential associations between hormone receptor genes and gender identity development (Theisen et al., 2019) but have been limited by small sample sizes. Although TIGR methodologies have advanced alongside genetic technologies, the field’s framing has changed less substantially, often retaining the older question about genetic determinants without incorporating contemporary social understandings of trans identity.

The proliferation of large biobanks and the increasing collection of gender identity data have created new opportunities to conduct TIGR, but little is known about how trans individuals view the possibility of researchers discovering a potential association, the risks and benefits of such research, or how researchers can meaningfully educate and engage trans people to support informed and community-based participation. Without this knowledge, it may be difficult for researchers to understand and prevent the potential harms of TIGR (*e.g.*, stigmatization, pathologization, medicalization, and eugenics) and to maximize its potential benefits (*e.g.*, personal validation, access to medical care, greater public acceptance, and increased support for advocacy), particularly when investigators lack the necessary experience in trans research; fail to fully consider the ethical, legal, and social implications (ELSIs) of their work; or fail to incorporate social, medical, and contextual factors that shape trans peoples’ lives (Rajkovic et al., 2022; Theisen et al., 2024; Hammack-Aviran et al., 2022). Existing studies on trans community perspectives of TIGR are limited as they are not always conducted in study populations consisting exclusively of trans participants. Qualitative research in this area underscores the need to quantitatively explore factors such as gender identity development, access to gender affirmation, and the broader social and political environment, given that these are central to how such research is interpreted (Rajkovic et al., 2022; Hammack-Aviran et al., 2022). One existing quantitative study (Theisen et al., 2024) developed a measure of attitudes toward transgender genomic research but did not include psychometric validation or modeling of correlates.

The rapid expansion of genomics research in recent years offers novel opportunities to understand human health, disease mechanisms, and potential therapeutic pathways (Brittain et al., 2017). The promise of genomics advancements raises important ELSI issues about how perceptions of marginalized identities are shaped by this research, how lived experiences are represented, and which questions are worthy of scientific investigation (Rebbeck et al., 2022; Popejoy and Fullerton, 2016). Ensuring inclusivity is essential for equity, empirical validity, and advancements in genomic medicine. Engaging trans communities meaningfully in research is not only crucial for realizing the full potential of genomics but also for safeguarding against perpetuating harm (Rajkovic et al., 2022; Levin et al., 2023; Polderman et al., 2018; Rebbeck et al., 2022; Cura et al., 2025).

The present study sought to quantitatively explore factors associated with trans individuals’ (1) perspectives on TIGR and (2) willingness to participate in TIGR.

## Materials and methods

### Research team

The Transforming Genomics: Maximizing Community Input and Impact Study sought to explore perspectives on TIGR and develop guidelines to inform future genomics research with trans populations using a sequential mixed-methods design consisting of qualitative interviews to inform a quantitative survey (the present study), sampling method, study procedures, survey instrument, data analyses, and dissemination. An executive stakeholder board (Board) comprising trans community members, advocates, clinicians, health equity scholars, and researchers co-led all aspects of the study. The investigative team met with the Board regularly throughout the study period to review study materials, provide feedback on research questions, guide recruitment, and review results and manuscripts. For the present analysis, we presented the results to Board members in a meeting, giving them an opportunity to ask questions and discuss their interpretations of the findings, which informed the writing of the *Discussion*. For example, Board members provided feedback on how to frame results, what ethical and political context to consider, and what prior research that should be included. Additionally, Board members were invited as co-authors, and those who accepted reviewed the final manuscript in detail and provided feedback.

The majority of individuals on our investigative team and Board identify as trans or queer, and members represent diversity across geography (within the United States), ethnoracial identity, age, gender identity, sexual orientation, prior experience in scientific research, fields of study (epidemiology, medicine, public health genetics, policy, and psychology), and genetics training. The primary analysts identify as trans and have training in epidemiology and public health genetics.

### Participants and procedures

Between January and May 2025, we recruited 495 trans participants using convenience sampling [a valuable method in transgender health research (Turban et al., 2023)] through professional, community, and social networks of the study’s Board and investigative team, including professional organizations, clinic populations, community-based organizations, and other professional and personal networks. Recruitment materials were not shared more broadly or online to prevent bots or fraudulent activity. Flyers instructed interested individuals to complete an eligibility screener on a secure data collection system to ensure that they met study eligibility criteria (18 years or older, lived in the U.S., and identified as trans). Previous experience or knowledge of genetics was not required for participation and we did not provide genetic education to participants prior to completing the survey. In collaboration with the Board, we established recruitment demographic quotas prior to survey launch, including recruiting more than 50% people of color and an even distribution of participants identifying as trans women, trans men, and nonbinary. Eligible individuals were purposively selected to complete a 30-min online survey to ensure maximum variation in the study population with regard to race, ethnicity, gender identity, sex assigned at birth, and geography.

Consistent with prior research by our team and others (Mozaffarian et al., 2025; Goodrich et al., 2023; Simone et al., 2024), we took precautions to ensure that individuals met the eligibility criteria and were not bots or fraudulent entries during screening and enrollment, such as manually comparing IP addresses to reported ZIP codes and reviewing open-ended responses (Theisen et al., 2024; Hammack-Aviran et al., 2022; Brittain et al., 2017). We also included an impossible (also known as honeypot) question—a survey question with illogical branching logic such that it is physically present in the code but hidden from human participants—as an additional bot protection measure (Morgan, 2024). Study activities were reviewed, approved, and determined to be exempt by the Institutional Review Boards at Mount Sinai and the University of Michigan.

### Measures

#### Outcome 1: perspectives on the ethical and societal impacts of TIGR

We abridged a 20-item scale developed by Theisen et al. (2024) to minimize response burden, creating a 14-item scale that included statements from each of five original subscales: beliefs on the origin of trans identity, personal effects, effects of TIGR on the trans community, effects of TIGR on society, and research team inclusivity and ethics. Participants could respond on a 5-point Likert scale (1 = strongly disagree to 5 = strongly agree). We added a question, “I believe that identifying as LGBTQ+ (lesbian, gay, bisexual, transgender, queer+) runs in families,” based on our qualitative findings, indicating that having an LGBTQ+ family member may influence perceptions of TIGR. Throughout the survey and manuscript, we use LGBTQ+ to refer to broader communities with diversity in sexual orientations and gender identity, and trans specifically when the construct or question is specific and exclusive to trans identities.

Using exploratory factor analysis (EFA) with a minimum residual factoring and oblique rotation to allow for correlated factors, we identified a three-factor solution with adequate item loadings from the short scale with three latent constructs: beliefs on the origins of gender identity, risks and benefits of TIGR, and research team composition (Supplementary Table 1). We summed responses into a continuous score (Cronbach’s alpha = 0.88) ranging from 14 to 70, with higher scores indicating more positive perspectives on TIGR. Correlation for individual items is shown in Supplementary Table 2.

#### Outcome 2: willingness to participate in TIGR

We created a single item for outcome 2: “I would be in a study to see if being trans and gender diverse is genetic” to be answered on a 5-point Likert scale and coded as disagree (1 = strongly disagree and 2 = disagree), unsure (3 = neither agree nor disagree), or agree (4 = agree and 5 = strongly agree). This coding structure was conceptually motivated by our interest in learning more about the unsure group and was empirically justified based on the sample distribution.

#### Sociodemographics and gender affirmation

We coded age categorically as 18-29 years old and ≥30 years old based on the median value. Individuals could select all that apply for ethnoracial identity. For regression analyses, we categorized participants into two groups [White vs. person of color (POC)] to increase sample sizes for statistical comparisons, and individuals who selected both White and another ethnoracial identity were categorized as POC. For gender identity, individuals could select nonbinary, transgender man, transgender woman, or another gender. Individuals who indicated their race was American Indian or Alaska Native were asked whether they identified as two-spirit (Government of Canada and C. I. of H. R., 2020). All participants were asked about their sex assigned at birth and whether they had a variation of sex characteristics (*i.e.*, intersex). We ascertained if participants reported a disability, either self-identified or via medical diagnosis (National Center for Women and Information Technology, 2021). We collected ZIP codes to categorize geographic location by U.S. Census region (Midwest, Northeast, Southeast, and West) (Census Bureau, 2017) and gender identity policy environment using a measure from the Movement Advancement Project (negative: indicating states with anti-trans policy environments; positive: indicating states with trans-friendly policy environments; and mixed: indicating states with both negative and positive trans policies). More information is included in Supplementary Material 3 (Movement Advancement Project, 2026). We assessed social gender affirmation (whether living full-time in their gender) (Deutsch et al., 2021), legal affirmation (whether identification documents and records list their affirmed name or gender) (Deutsch et al., 2021), and medical affirmation (whether they have ever sought out medical care to transition or affirm their gender) (Deutsch et al., 2021).

#### Social determinants of health

We collected education, health insurance, employment status, annual individual income, food insecurity, housing insecurity, and worry about losing housing.

#### Policy context

We asked two questions to assess policy awareness and sense of safety (Restar et al., 2024): (1) “How familiar are you with legislation/bills in the United States that use binary sex/gender to purposefully restrict trans peoples’ rights?” and (2) “How safe do you feel in the current policy environment around transgender issues in your state?” Participants could respond on a 5-point Likert scale (1 = extremely safe to 5 = not at all safe). Responses were dichotomized at the median as “Very familiar” (2 = very familiar and 1 = extremely familiar) and “Less familiar” (5 = not at all familiar, 4 = slightly familiar, and 3 = moderately familiar) for the first question and as “More safe” (3 = moderately safe, 2 = very safe, and 1 = extremely safe) and “Less safe” (5 = not at all safe and 4 = slightly safe) for the second question. These cut points were selected due to small cell sizes and to ensure sufficient statistical power.

#### Healthcare

We measured medical mistrust via a single item (Shea et al., 2008) on a 5-point Likert scale (1 = strongly disagree to 5 = strongly agree): “All patients get the same medical treatment in the healthcare system, no matter the patient’s gender identity,” dichotomized at the median into low (>1, indicating individuals disagreed, neither agreed nor disagreed, agreed, or strongly agreed with the statement) and high (=1, indicating individuals strongly disagreed with the statement) medical mistrust. Healthcare empowerment was captured by a single item using the same Likert scale: “I feel confident I can advocate for myself to get the healthcare I deserve,” dichotomized as low (≤4, indicating individuals agreed, neither agreed nor disagreed, disagreed, or strongly disagreed with the statement) and high (>4, indicating individuals strongly agreed with the statement). To assess healthcare access, participants were asked, “In the last 12 months, have you avoided or delayed getting healthcare you needed when injured or sick due to fear of mistreatment due to being a transgender or gender-diverse person” (National Health Interview Survey, 2024), to which individuals could respond “Yes, one time,” “Yes, more than one time,” or “No.” Responses were dichotomized into binary yes/no.

#### Physical health

We asked a single item from the 12-item Short-Form Health Survey (SF-12): “In general, how would you say your health is?” Responses were dichotomized into good (1 = excellent, 2 = very good, and 3 = good) and poor (4 = fair and 5 = poor).

#### Prior research participation

Participants were asked whether they had previously participated in a medical research study or a research study that involved genetic testing. Response options for both questions were dichotomized to yes/no.

### Data analysis

We calculated descriptive statistics for the overall sample and stratified by level of willingness to participate in TIGR. Chi-square or Fisher’s exact tests (for cell sizes with <5 observations) assessed differences in sample characteristics by willingness level. For the TIGR scale (first outcome), we computed the distribution of each item. Data were assumed missing at random, and missingness ranged from 0.40% to 5.66% across survey items. We used multiple imputation (five imputations) by chained equations to address missingness (Buuren et al., 2011). We fit bivariate and multivariable regression models to the imputed data and pooled parameter estimates using Rubin’s rules (Rubin, 2009). We included variables that were significantly associated with the outcome at the bivariate level (*p* < 0.05) in the final multivariable model for that outcome to improve model parsimony with our limited sample size. Prior to modeling, we assessed assumptions to ensure normality (for linear models), linearity in the logit (for multinomial models), and absence of multicollinearity (variance inflation factor <2.0 for all models).

For outcome 1, we fit linear regression models to assess each variable’s association with TIGR perspectives (continuous scale scores). We z-scored (mean = 0, SD = 1) TIGR perspective scores to facilitate interpretation as standard deviation unit changes in the statistical predictor relative to the outcome (standardized slopes ranging from −1.0 to 1.0). Models estimated crude β, adjusted β (aβ), and 95% confidence intervals (95% CI). For outcome 2, we fit multinomial logistic regression models to estimate each variable’s association with TIGR research participation (agree, unsure, or disagree). We fit two multivariable models—one with and one without TIGR scale scores—to examine the change in the relationship between the independent and dependent variables with the inclusion of perspectives on TIGR and to assess the potential mediating role of TIGR perspectives. Given the cross-sectional study design, we did not perform formal mediation analyses. Parameter estimates were exponentiated to generate crude odds ratios (OR), adjusted OR (aOR), and 95% CI. The reference category for the multinomial models was disagree. All analyses were conducted using R version 4.4.1 with two-sided p-values and statistical significance established at α = 0.05.

## Results

### Descriptive statistics

Descriptive characteristics of the sample are presented in Table 1. Among the 495 participants, the median age was 29.0 years (IQR = 25–34; mean = 30.8; SD = 9.4), and the majority were White (N = 303/492, 61.6%). Thirty-seven percent (36.8%) were nonbinary, 32.3% transgender men, and 26.1% transgender women; most participants were assigned female at birth (66.9%). Fifty-three percent (52.9%) indicated they had a disability (most commonly a mental health condition, attention deficit disorder, or autism). More than half (54.6%) came from positive policy environments and 28% from negative policy environments. Most participants had graduated from college (67.1%), were employed full-time (53.1%), and earned $25,000–$74,999 annually (46.3%).

**TABLE 1:** Descriptive statistics for the overall study sample of transgender, nonbinary, and gender diverse adults in the United States (n = 495).

Participant characteristics	Total study sample (n = 495)		I would be in a study to see if being trans and gender diverse is genetic						*p*-value^a^
			Disagree (n = 87)		Unsure (n = 100)		Agree (n = 308)		
	n	%	n	%	n	%	n	%	
**Age**	​	​	​	​	​	​	​	​	0.5614
Mean (SD) Median (IQR)	30.8 (9.4) 29.0 (25.0–34.0)		31.4 (7.1) 30.0 (26.0–35.0)		30.8 (10.5) 28.5 (25.0–34.3)		29.0 (9.5) 29.0 (25.0–34.0)		​
18–29 years old	254	51.3%	41	47.1%	55	55.0%	158	51.3%	​
≥30 years old	241	48.7%	46	52.9%	45	45.0%	150	48.7%	​
**Ethnoracial identity^b^ ^c^**	​	​	​	​	​	​	​	​	**0.0001**
People of color	189	38.2%	17	19.5%	36	36.0%	136	44.2%	​
American Indian or Alaska Native	11	2.2%	0	0.0%	4	4.0%	7	2.3%	​
Asian	47	9.5%	7	8.1%	3	3.0%	37	12.0%	​
Black or African American	67	13.5%	4	4.6%	16	16.0%	47	15.3%	​
Hispanic or Latino/a/x/e	68	13.7%	5	5.8%	17	17.0%	46	14.9%	​
Middle Eastern or North African	13	2.6%	2	2.3%	2	2.0%	9	2.9%	​
Native Hawaiian or Pacific Islander	3	0.6%	0	0.0%	0	0.0%	3	1.0%	​
White	363	73.3%	75	86.2%	71	71.0%	217	70.5%	​
Another racial/ethnic identity	13	2.6%	2	2.3%	3	3.0%	8	2.6%	​
Choose not to answer	3	0.6%	0	0.0%	1	1.0%	2	0.7%	​
Gender identity									
Nonbinary	182	36.8%	36	41.4%	39	39.0%	107	34.7%	0.1623
Transgender man	160	32.3%	33	37.9%	31	31.0%	96	31.2%	​
Transgender woman	129	26.1%	14	16.1%	25	25.0%	90	29.2%	​
Another gender identity	23	4.7%	4	4.6%	5	5.0%	14	4.6%	​
Choose not to answer	1	0.2%	0	0.0%	0	0.0%	1	0.3%	​
**Two-spirit^d^**	​	​	​	​	​	​	​	​	0.4643
No	3	27.3%	0	0.0%	0	0.0%	3	1.0%	​
Yes	6	54.6%	0	0.0%	3	3.0%	3	1.0%	​
Choose not to answer	2	18.2%	0	0.0%	1	1.0%	1	0.3%	​
**Sex assigned at birth**	​	​	​	​	​	​	​	​	**0.0268**
Female	331	66.9%	68	78.2%	69	69.0%	194	63.0%	​
Male	159	32.1%	18	20.7%	31	31.0%	110	35.7%	​
Another sex	1	0.2%	0	0.0%	0	0.0%	1	0.3%	​
Choose not to answer	4	0.8%	1	1.2%	0	0.0%	3	1.0%	​
**Intersex**	​	​	​	​	​	​	​	​	0.0727
No	373	75.4%	74	85.1%	78	78.0%	221	71.8%	​
Yes	12	2.4%	1	1.2%	0	0.0%	11	3.6%	​
I do not know	108	21.8%	12	13.8%	22	22.0%	74	24.0%	​
Choose not to answer	2	0.4%	0	0.0%	0	0.0%	2	0.7%	​
**Disability status**									0.6530
No	225	45.5%	35	40.2%	48	48.0%	142	46.1%	​
Yes^e^	262	52.9%	48	55.2%	50	50.0%	164	53.3%	​
Choose not to answer	8	1.6%	4	4.6%	2	2.0%	2	0.7%	​
Geographical location	​	​	​	​	​	​	​	​	0.4918
Midwest	57	11.5%	6	6.9%	15	15.0%	36	11.7%	​
Northeast	192	38.8%	38	43.7%	39	39.0%	115	37.3%	​
Southeast	147	29.7%	21	24.1%	30	30.0%	96	31.2%	​
West	88	17.8%	16	18.4%	14	14.0%	58	18.8%	​
Choose not to answer	11	2.2%	6	6.9%	2	2.0%	2	0.7%	​
**Education**	​	​	​	​	​	​	​	​	**0.0005**
Completed high school/GED or less	43	8.7%	2	2.3%	9	9.0%	32	10.4%	​
Some college or associate degree	120	24.2%	11	12.6%	21	21.0%	88	28.6%	​
College/bachelor’s degree or more	332	67.1%	74	85.1%	70	70.0%	188	61.0%	​
**Health insurance**	​	​	​	​	​	​	​	​	0.1763
Private	332	67.1%	64	73.6%	72	72.0%	196	63.6%	​
Other	141	28.5%	22	25.3%	23	23.0%	96	31.2%	​
I do not know	14	2.8%	1	1.2%	5	5.0%	8	2.6%	​
Choose not to answer	8	1.6%	0	0.0%	0	0.0%	8	2.6%	​
**Employment**	​	​	​	​	​	​	​	​	0.0603
Employed part-time	120	24.2%	14	16.1%	26	26.0%	80	26.0%	​
Employed full-time	263	53.1%	58	66.7%	52	52.0%	153	49.7%	​
Unemployed	102	20.6%	12	13.8%	22	22.0%	68	22.1%	​
Choose not to answer	10	2.0%	3	3.5%	0	0.0%	7	2.3%	​
**Annual individual income**	​	​	​	​	​	​	​	​	0.2304
≤ $24,999	141	28.5%	22	25.3%	28	28.0%	91	29.6%	​
$25,000–$74,999	229	46.3%	36	41.4%	52	52.0%	141	45.8%	​
≥ $75,000	97	19.6%	24	27.6%	15	15.0%	58	18.8%	​
I do not know	8	1.6%	0	0.0%	3	3.0%	5	1.6%	​
Choose not to answer	20	4.0%	5	5.8%	2	2.0%	13	4.2%	​
**Food insecurity**	​	​	​	​	​	​	​	​	**< 0.0001**
Do not run out of food	270	54.6%	62	71.3%	67	67.0%	141	45.8%	​
Ever run out of food	215	43.4%	23	26.4%	32	32.0%	160	52.0%	​
Choose not to answer	10	2.0%	2	2.3%	1	1.0%	7	2.3%	​
**Housing insecurity**	​	​	​	​	​	​	​	​	0.0994
Never homeless	338	68.3%	64	73.6%	76	76.0%	198	64.3%	​
Homeless ever	113	22.8%	19	21.8%	16	16.0%	78	25.3%	​
Homeless in the past 12 months	31	6.3%	2	2.3%	6	6.0%	23	7.5%	​
Choose not to answer	13	2.6%	2	2.3%	2	2.0%	9	2.9%	​
**Worry over losing housing**	​	​	​	​	​	​	​	​	0.2888
No	332	67.1%	63	72.4%	70	70.0%	199	64.6%	​
Yes	150	30.3%	22	25.3%	27	27.0%	101	32.8%	​
Choose not to answer	13	2.6%	2	2.3%	3	3.0%	8	2.6%	​
**Social affirmation**	​	​	​	​	​	​	​	​	0.7301
No	110	22.2%	17	19.5%	24	24.0%	69	22.4%	​
Yes	378	76.4%	70	80.5%	75	75.0%	233	75.7%	​
Choose not to answer	7	1.4%	0	0.0%	1	1.0%	6	1.95%	​
**Legal affirmation**	​	​	​	​	​	​	​	​	0.9223
None of my IDs and records list my affirmed name or gender	142	28.7%	25	28.7%	31	31.0%	86	27.9%	​
Any of my IDs and records list my affirmed name or gender	326	65.9%	58	66.7%	62	62.0%	206	66.9%	​
I do not wish to change my name or gender	20	4.0%	3	3.5%	5	2.0%	12	3.9%	​
Choose not to answer	7	1.4%	1	1.2%	2	2.0%	4	1.3%	​
Medical affirmation^f^	​	​	​	​	​	​	​	​	​
No	100	20.2%	12	13.8%	25	25.0%	63	20.5%	​
Yes	395	79.8%	75	86.2%	75	75.0%	245	79.6%	​
Choose not to answer	0	0.0%	0	0.0%	0	0.0%	0	0.0%	​
**Policy environment^g^**	​	​	​	​	​	​	​	​	**0.0482**
Negative	139	28.1%	13	14.9%	33	33.0%	93	30.2%	​
Mixed	75	15.2%	14	16.1%	18	18.0%	43	14.0%	​
Positive	270	54.6%	54	62.1%	47	47.0%	169	54.9%	​
Choose not to answer	11	2.2%	6	6.9%	2	2.0%	2	0.7%	​
Policy awareness	​	​	​	​	​	​	​	​	​
Very familiar	334	67.5%	70	80.5%	57	57.0%	207	67.2%	​
Less familiar	159	32.1%	17	19.5%	43	43.0%	99	32.1%	​
I do not know	2	0.4%	0	0.0%	0	0.0%	2	0.7%	​
**Policy safety**	​	​	​	​	​	​	​	​	0.8378
Safe	194	39.2%	35	40.2%	36	36.0%	123	39.9%	​
Unsafe	294	59.4%	52	59.8%	61	61.0%	181	58.8%	​
I do not know	6	1.2%	0	0.0%	2	2.0%	4	1.3%	​
Choose not to answer	1	0.2%	0	0.0%	1	1.0%	0	0.0%	​
**Medical mistrust**	​	​	​	​	​	​	​	​	**< 0.0001**
Low	129	26.1%	5	5.8%	32	32.0%	92	29.9%	​
High	366	73.9%	82	94.3%	68	68.0%	216	70.1%	​
**Healthcare empowerment**	​	​	​	​	​	​	​	​	0.2555
Low	387	78.2%	68	78.2%	80	80.0%	239	77.6%	​
High	108	21.8%	19	21.8%	20	20.0%	69	22.4%	​
**Healthcare avoidance**	​	​	​	​	​	​	​	​	**0.0169**
No	277	56.0%	60	69.0%	58	58.0%	159	51.6%	​
Yes	213	43.0%	27	31.0%	40	40.0%	146	47.4%	​
Choose not to answer	5	1.0%	0	27.6%	2	2.0%	3	1.0%	​
**Physical health**	​	​	​	​	​	​	​	​	0.1079
Good	338	78.4%	63	72.4%	85	85.0%	240	77.9%	​
Poor	107	21.6%	24	27.6%	15	15.0%	68	22.1%	​
**Participation in medical research**	​	​	​	​	​	​	​	​	0.0752
No	230	48.5%	30	34.5%	50	50.0%	150	48.7%	​
Yes	218	44.0%	46	52.9%	42	42.0%	130	42.2%	​
I do not know	46	9.3%	10	11.5%	8	8.0%	28	9.1%	​
Choose not to answer	1	0.2%	1	1.2%	0	0.0%	0	0.0%	​
**Participation in genomic research**	​	​	​	​	​	​	​	​	0.7011
No	439	88.7%	80	92.0%	90	90.0%	269	87.3%	​
Yes	43	8.7%	6	6.9%	8	8.0%	29	9.4%	​
I do not know	13	2.6%	1	1.2%	2	2.0%	10	3.3%	​

^a^ *p*-values were obtained from chi-square statistical tests; Fisher’s exact tests were used for cell sizes <5. “I do not know,” “Other,” and “Choose not to answer” were not included in statistical tests. Bolded values indicate statistical significance at the *p* < 0.05 level.

^b^ Participants could select all responses that apply, and individuals who selected both White and another ethnoracial identity were categorized as POC. Sum of % may be greater than 100%.

^c^ Responses were grouped to increase sample sizes for statistical analyses.

^d^ Participants were only shown this question if they indicated their ethnoracial identity as “American Indian or Alaska Native” (% denominator = 11).

^e^ Self-reported disabilities were as follows: 56.5% attention deficit, 50.8% autism, 29.0% health-related disability, 12.6% mobility-related disability, 11.8% learning disability, 5.0% deaf/hard of hearing, 3.8% blind/visually impaired, 1.5% speech-related disability, and 6.9% other [denominator for specific disability conditions % is equal to the number of participants who indicated that they had a disability (n = 262)]. Participants could select all responses that apply; sum of % may be greater than 100%. Specific disability conditions were not included in the chi-square test.

^f^ Medical affirmation includes any kind of medical gender affirmation to transition or affirm an individual gender, including hormones, surgery, and electrolysis, among others.

^g^ Policy categories defined by the Movement Advancement Project, ranging from “Negative” (indicating states with more anti-trans policy environments) to “Positive” (indicating states with trans-friendly policy environments). “Mixed” indicated states with a mix of both negative and positive trans policies.

^h^ Psychological distress measured using the Kessler-6 validated cut-point for serious psychological distress (score ≥13).

Most participants lived full-time in their gender identity (76.4%) and had sought medical gender affirmation (79.8%); 28.7% had no legal affirmation. More than half were very familiar with anti-trans policies that use binary definitions of sex or gender (67.5%) and felt less safe in the current political environment (59.4%). There was a high level of medical mistrust (74.6%). Most (78.4%) self-reported good physical health, and 58.8% reported serious psychological distress. Although 44% had participated in medical research, only 8.7% had participated in research involving genetic testing.

### Outcome 1—TIGR scale scores

**FIGURE 1:**
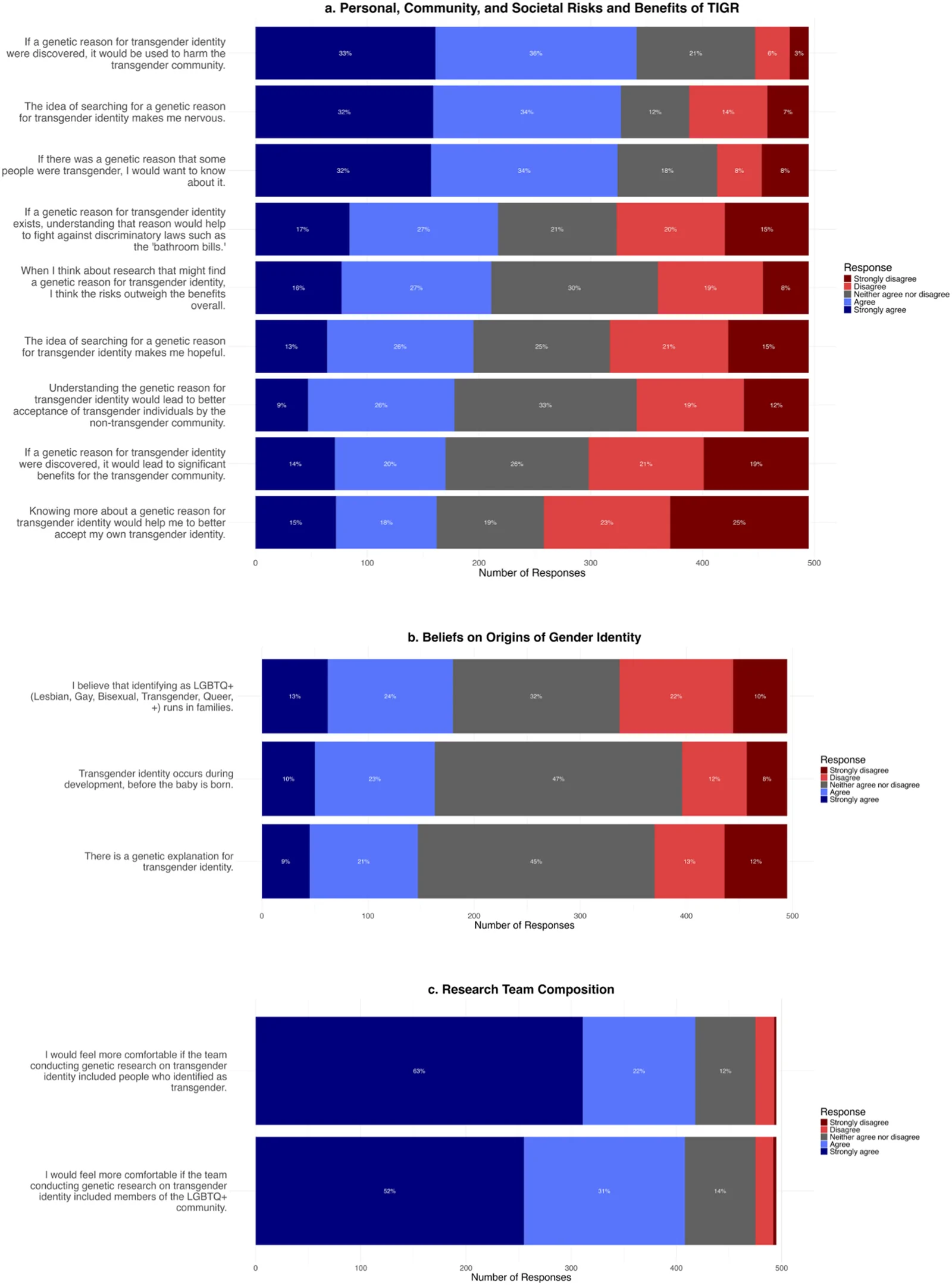
Descriptive statistics for the transgender identity genomics research (TIGR) scale. **(a)** Personal, community, and societal risks and benefits of TIGR subscale. **(b)** Beliefs on origins of gender identity subscale. **(c)** Research team composition subscale. For Item 1 (“I would feel more comfortable if the team conducting genetic research on transgender identity included people who identified as transgender.”), values for disagree and strongly disagree are 3.4% and 0.6%, respectively. For Item 2 (“I would feel more comfortable if the team conducting genetic research on transgender identity included members of the LGBTQ+ community.”), values for disagree and strongly disagree are 3.6% and 0.4%, respectively.

Descriptive statistics for TIGR subscales are presented in Table 2 and Figure 1. The mean summed score was 43.7 (SD = 10.1; range = 14–70, with higher scores indicating more positive perspectives on TIGR). There was a high level of uncertainty for many items, particularly for the ‘Beliefs on origins of gender identity’ factor, with 31.7%–47.1% of individuals indicating that they neither agreed nor disagreed with each item. Many individuals perceived potential benefits of TIGR at the personal and societal levels. The majority (65.4%) agreed that they would want to know whether there was a genetic reason that some people are trans; 32.8% agreed that knowing more about a potential explanation for trans identity would help them better accept their trans identity; and 43.9% agreed that such a finding could be used to help fight discriminatory laws. Still, most participants (66.0%) agreed that searching for a genetic explanation for trans identity makes them nervous, and 68.9% agreed that TIGR could be used to harm trans communities. Most participants agreed that they would feel more comfortable if the research teams conducting TIGR contained trans (84.4%) or LGBTQ+ community members (82.4%).

**TABLE 2:** Descriptive statistics for the trans identity genomic research (TIGR) scale.

Item	Mean (SD)	Strongly disagree (n)	%	Disagree (n)	%	Neither agree nor disagree (n)	%	Agree (n)	%	Strongly agree (n)	%
Beliefs on origins of gender identity											
There is a genetic explanation for transgender identity	3.0 (1.1)	59	11.9%	66	13.3%	223	45.1%	102	20.6%	45	9.1%
I believe that identifying as LGBTQ+ (lesbian, gay, bisexual, transgender, queer, +) runs in families	3.0 (1.2)	51	10.3%	107	21.6%	157	31.7%	118	23.8%	62	12.5%
Transgender identity occurs during development, before the baby is born	3.2 (1.0)	38	7.7%	61	12.3%	233	47.1%	113	22.8%	50	10.1%
Personal, community, and societal risks and benefits of TIGR											
If there was a genetic reason that some people were transgender, I would want to know about it	3.7 (1.2)	42	8.5%	40	8.1%	89	18.0%	167	33.7%	157	31.7%
The idea of searching for a genetic reason for transgender identity makes me nervous	3.7 (1.3)	37	7.5%	70	14.1%	61	12.3%	168	33.9%	159	32.1%
The idea of searching for a genetic reason for transgender identity makes me hopeful	3.0 (1.3)	72	14.6%	106	21.4%	122	24.7%	131	26.5%	64	12.9%
Knowing more about a genetic reason for transgender identity would help me better accept my own transgender identity	2.7 (1.4)	124	25.0%	113	22.8%	96	19.4%	90	18.2%	72	14.6%
If a genetic reason for transgender identity were discovered, it would lead to significant benefits for the transgender community	2.9 (1.3)	94	19.0%	103	20.8%	128	25.9%	99	20.0%	71	14.3%
If a genetic reason for transgender identity were discovered, it would be used to harm the transgender community	3.9 (1.0)	17	3.4%	31	6.3%	106	21.4%	180	36.4%	161	32.5%
Understanding the genetic reason for transgender identity would lead to better acceptance of transgender individuals by the non-transgender community	3.0 (1.1)	58	11.7%	96	19.4%	163	32.9%	131	26.5%	47	9.5%
If a genetic reason for transgender identity exists, understanding that reason would help fight against discriminatory laws such as the “bathroom bills”	3.1 (1.3)	75	15.2%	97	19.6%	106	21.4%	133	26.9%	84	17.0%
When I think about research that might find a genetic reason for transgender identity, I think the risks outweigh the benefits overall	3.2 (1.2)	41	8.3%	94	19.0%	149	30.1%	134	27.1%	77	15.6%
Research team composition											
I would feel more comfortable if the team conducting genetic research on transgender identity included members of the LGBTQ+ community	4.3 (0.9)	3	0.6%	17	3.4%	67	13.5%	153	30.9%	255	51.5%
I would feel more comfortable if the team conducting genetic research on transgender identity included people who were identified as transgender	4.4 (0.9)	2	0.4%	18	3.6%	57	11.5%	107	21.6%	311	62.8%

Bivariate and multivariable linear regression models for TIGR scale scores are displayed in Table 3. In the multivariable model, POC vs. White individuals (aβ = 0.48, 95% CI = 0.18 to 0.56) and individuals with food insecurity vs. not (aβ = 0.21, 95% CI = 0.02 to 0.40) had more positive views on TIGR. Groups with less favorable views of TIGR were individuals with disabilities vs. those without (aβ = −0.20, 95% CI = −0.38 to −0.02), those who received medical affirmation vs. those who did not (aβ = −0.30, 95% CI = −0.54 to −0.06), individuals residing in mixed vs. negative trans policy environments (aβ = −0.32, 95% CI = −0.59 to −0.05), and individuals with high vs. low medical mistrust (aβ = −0.24, 95% CI = −0.46 to −0.03).

**TABLE 3:** Bivariate and multivariable linear regression models: correlates of transgender identity genomics research (TIGR) scale scores.^a^.

Coefficient	Bivariate			Multivariable		
	β	95% CI	*p*-value	aβ	95% CI	*p*-value
Age (ref: 18–29 years)						
≥30 years	0.03	−0.15, 0.21	0.7456	​	​	​
Race (ref: White)						
People of color	0.57	0.40, 0.75	**<0.0001**	0.48	0.18, 0.56	**0.0002**
Gender identity (ref: nonbinary)						
Transgender man	0.14	−0.07, 0.35	0.1949	0.15	−0.06, 0.37	0.1640
Transgender woman	0.46	0.24, 0.68	**<0.0001**	0.24	−0.00, 0.48	0.0528
Disability status (ref: no)						
Yes	−0.3	−0.48, −0.13	**0.0007**	−0.20	−0.38, −0.02	**0.0351**
Geographical location (ref: Midwest)						
Northeast	−0.04	−0.34, 0.25	0.7818	​	​	​
Southeast	0.28	−0.03, 0.58	0.0726	​	​	​
West	0.21	−0.12, 0.54	0.2215	​	​	​
Education (ref: completed high school/GED or less)						
Some college or associate degree	−0.23	−0.57, 0.11	0.1926	0.05	−0.29, 0.40	0.7669
Completed college/bachelor’s degree or more	−0.63	−0.94, −0.33	**<0.0001**	−0.16	−0.49, 0.17	0.3385
Insurance (ref: private)						
Public/TRICARE/VA/Indian health service/no insurance	0.18	−0.01, 0.38	0.0651	​	​	​
Employment (ref: employed part-time)						
Employed full-time	−0.10	−0.32, 0.11	0.3489	​	​	​
Unemployed	0.06	−0.21, 0.32	0.6783	​	​	​
Income (ref: < $24,999)						
$25,000–$74,999	−0.03	−0.24, 0.19	0.8171	​	​	​
≥ $75,000	−0.26	−0.52, 0.00	0.0537	​	​	​
Food insecurity (ref: do not run out of food)						
Ever run out of food	0.43	0.25, 0.60	**<0.0001**	0.21	0.02, 0.40	**0.0276**
Housing insecurity (ref: never experienced homelessness)						
Experienced homelessness ever	0.12	−0.09, 0.32	0.2790	​	​	​
Experienced homelessness in the past 12 months	0.35	−0.01, 0.72	0.0593	​	​	​
Worry over losing housing (ref: no)						
Yes	0.19	−0.00, 0.38	0.0534	​	​	​
Social affirmation (ref: no)						
Yes	−0.08	−0.30, 0.13	0.4396	​	​	​
Legal affirmation (ref: none)						
At least some my IDs and records list my affirmed name or gender	0.01	−0.19, 0.20	0.9602	​	​	​
I do not wish to change my name or gender	−0.25	−0.72, 0.21	0.2871	​	​	​
Medical affirmation (ref: no)						
Yes	−0.26	−0.48, −0.04	**0.0191**	−0.30	−0.54, −0.06	**0.0133**
Policy environment (ref: negative)						
Mixed	−0.41	−0.69, −0.13	**0.0041**	−0.32	−0.59, −0.05	**0.0221**
Positive	−0.30	−0.50, −0.10	**0.0039**	−0.19	−0.39, 0.02	0.0729
Policy awareness (ref: very familiar)						
Less familiar	0.24	0.06, 0.43	**0.0112**	0.15	−0.03, 0.34	0.1072
Policy safety (ref: more safe)						
Less safe	0.05	−0.13, 0.23	0.5979	​	​	​
Medical mistrust (ref: low)						
High	−0.58	−0.78, −0.38	**<0.0001**	−0.24	−0.46, −0.03	**0.0276**
Healthcare empowerment (ref: low)						
High	0.27	0.06, 0.48	**0.0140**	0.09	−0.12, 0.30	0.4000
Healthcare avoidance (ref: no)						
Yes	0.14	−0.03, 0.32	0.1118	​	​	​
General health (ref: excellent/very good/good)						
Fair/poor	−0.08	−0.30, 0.13	0.4536	​	​	​
Participation in medical research (ref: no)						
Yes	−0.23	−0.41, −0.06	**0.0101**	−0.17	−0.35, 0.01	0.0581
Participation in genomic research (ref: no)						
Yes	0.08	−0.20, 0.36	0.5714	​	​	​

^a^ β, beta; Aβ, adjusted beta; 95% CI, 95% confidence interval. TIGR scores were z-scores (mean = 0, SD = 1) and interpretable as standard deviation unit changes in the statistical predictor relative to the outcome (standardized slopes ranging from −1.0 to 1.0). Bolded values indicate statistical significance at the *p* < 0.05 level.

### Outcome 2—willingness to participate in TIGR

Among participants, most (62.2%) reported being willing to participate in TIGR (*i.e.*, agreement), while 20.2% were unsure, and 17.6% were unwilling to participate in such studies (*i.e.*, disagreement; the referent level). Bivariate and multivariable multinomial logistic regression models for willingness to participate in TIGR are displayed in Table 4. In multivariable model 1, we included all variables that reached statistical significance in bivariate analyses. In multivariable model 2, we included these variables in addition to the three TIGR scale factors (beliefs on the origins of gender identity; personal, community, and societal risks and benefits of TIGR; and research team composition).

**TABLE 4:** Bivariate and multivariable multinomial logistic regression models: intention to participate in transgender identity genomic research (TIGR) (ref: disagree).^a^

Coefficient	Bivariate						Multivariable model 1, no TIGR						Multivariable model 2, added TIGR					
	Unsure (n = 100)			Agree (n = 308)			Unsure (n = 100)			Agree (n = 308)			Unsure (n = 100)			Agree (n = 308)		
	OR	95% CI	*p*-value	OR	95% CI	*p*-value	aOR	95% CI	*p*-value	aOR	95% CI	*p*-value	aOR	95% CI	*p*-value	aOR	95% CI	*p*-value
Age (ref: 18–29 years)																		
≥30 years	0.73	0.41, 1.30	0.2833	0.85	0.53, 1.36	0.4921	​	​	​	​	​	​	​	​	​	​	​	​
Ethnoracial identity (ref: White)																		
People of color	2.35	1.20, 4.60	**0.0126**	3.29	1.85, 5.80	**<0.0001**	2.50	1.13, 5.56	**0.0248**	2.73	1.35, 5.53	**0.0055**	1.56	0.61, 4.00	0.3561	1.19	0.47, 3.01	0.7062
Gender identity (ref: nonbinary)	​	​	​	​	​	​	​	​	​	​	​	​	​	​	​	​	​	​
Transgender man	0.87	0.44, 1.69	0.6757	0.98	0.57, 1.69	0.9387	0.71	0.33, 1.50	0.3679	0.87	0.47, 1.63	0.6708	0.75	0.31, 1.79	0.5107	0.76	0.32, 1.79	0.5340
Transgender woman	1.65	0.74, 3.65	0.2189	2.16	1.10, 4.26	**0.0262**	1.26	0.50, 3.16	0.6292	1.42	0.64, 3.18	0.3880	1.50	0.51, 4.41	0.4628	1.31	0.45, 3.80	0.6182
Disability status (ref: no)																		
Yes	0.76	0.42, 1.37	0.3606	0.84	0.52, 1.38	0.4935	​	​	​	​	​	​	​	​	​	​	​	​
Geographical location (ref: Midwest)																		
Northeast	0.41	0.14, 1.17	0.0962	0.50	0.20, 1.29	0.1537	​	​	​	​	​	​	​	​	​	​	​	​
Southeast	0.57	0.19, 1.72	0.3187	0.76	0.29, 2.04	0.5886	​	​	​	​	​	​	​	​	​	​	​	​
West	0.35	0.11, 1.15	0.0839	0.60	0.22, 1.69	0.3363	​	​	​	​	​	​	​	​	​	​	​	​
Education (ref: completed high school/GED or less)																		
Some college or associate degree	0.42	0.08, 2.32	0.3225	0.50	0.11, 2.38	0.3841	0.63	0.10, 3.87	0.6208	1.05	0.20, 5.59	0.9542	0.38	0.04, 3.71	0.4041	0.62	0.06, 5.97	0.6778
College/bachelor’s degree or more	0.21	0.11, 2.38	0.0516	0.16	0.04, 0.68	**0.0134**	0.51	0.09, 2.78	0.4350	0.65	0.13, 3.12	0.5875	0.57	0.07, 4.86	0.6053	0.94	0.11, 8.00	0.9541
Health insurance (ref: private)																		
Other	0.93	0.47, 1.82	0.8314	1.42	0.83, 2.45	0.2015	​	​	​	​	​	​	​	​	​	​	​	​
Employment (ref: employed part-time)																		
Employed full-time	0.48	0.23, 1.02	0.0576	0.46	0.24, 0.88	**0.0187**	0.58	0.25, 1.36	0.2082	0.75	0.36, 1.58	0.4456	0.40	0.14, 1.12	0.0807	0.41	0.15, 1.14	0.0887
Unemployed	0.99	0.38, 2.57	0.9789	0.99	0.43, 2.29	0.9747	1.28	0.42, 3.94	0.6650	1.23	0.45, 3.36	0.6839	1.07	0.29, 3.99	0.9247	0.99	0.27, 3.66	0.9906
Income (ref: < $24,999)																		
$25,000–$74,999	1.14	0.56, 2.29	0.7238	0.95	0.52, 1.71	0.8568	​	​	​	​	​	​	​	​	​	​	​	​
≥ $75,000	0.49	0.21, 1.15	0.1030	0.58	0.30, 1.14	0.1143	​	​	​	​	​	​	​	​	​	​	​	​
Food insecurity (ref: do not run out of food)																		
Ever run out of food	1.29	0.68, 2.44	0.4377	3.06	1.80, 5.19	**<0.0001**	1.02	0.47, 2.25	0.9529	2.24	1.16, 4.33	**0.0172**	0.72	0.28, 1.83	0.4890	1.59	0.65, 3.86	0.3087
Housing insecurity (ref: never homeless)																		
Ever homeless	0.72	0.35, 1.48	0.3725	1.32	0.76, 2.31	0.3289	​	​	​	​	​	​	​	​	​	​	​	​
Homeless in the past 12 months	2.40	0.46, 12.53	0.2992	3.73	0.85, 16.31	0.0820	​	​	​	​	​	​	​	​	​	​	​	​
Worry over losing housing (ref: no)																		
Yes	1.10	0.57, 2.13	0.7681	1.45	0.85, 2.50	0.1768	​	​	​	​	​	​	​	​	​	​	​	​
Social affirmation (ref: no)																		
Yes	0.76	0.38, 1.53	0.4413	0.82	0.45, 1.49	0.5132	​	​	​	​	​	​	​	​	​	​	​	​
Legal affirmation (ref: none)																		
At least some of my IDs and records list my affirmed name or gender	0.86	0.46, 1.63	0.6481	1.03	0.61, 1.76	0.9064	​	​	​	​	​	​	​	​	​	​	​	​
I do not wish to change my name or gender	1.34	0.29, 6.18	0.7041	1.16	0.30, 4.45	0.8257	​	​	​	​	​	​	​	​	​	​	​	​
Medical affirmation (ref: no)																		
Yes	0.48	0.23, 1.03	0.0587	0.62	0.32, 1.22	0.1654	​	​	​	​	​	​	​	​	​	​	​	​
Policy environment (ref: negative)																		
Mixed	0.51	0.20, 1.31	0.1604	0.43	0.19, 0.99	**0.0483**	0.70	0.25, 1.99	0.5029	0.55	0.22, 1.39	0.2100	0.85	0.25, 2.93	0.7971	0.96	0.28, 3.32	0.9468
Positive	0.34	0.16, 0.73	**0.0055**	0.44	0.23, 0.84	**0.0139**	0.51	0.22, 1.18	0.1162	0.66	0.32, 1.39	0.2736	0.52	0.19, 1.42	0.2048	0.92	0.34, 2.47	0.8658
Policy awareness (ref: very familiar)																		
Less familiar	3.11	1.60, 6.02	**0.0008**	1.97	1.10, 3.52	**0.0228**	2.56	1.21, 5.41	**0.0142**	1.60	0.82, 3.15	0.1711	1.47	0.61, 3.52	0.3925	0.76	0.32, 1.93	0.5462
Policy safety (ref: more safe)																		
Less safe	1.14	0.63, 2.07	0.6636	0.99	0.61, 1.61	0.9705	​	​	​	​	​	​	​	​	​	​	​	​
Medical mistrust (ref: low)																		
High	0.14	0.05, 0.39	**0.0002**	0.15	0.06, 0.37	**<0.0001**	0.24	0.08, 0.68	**0.0080**	0.26	0.10, 0.69	**0.0075**	0.43	0.13, 1.42	0.1639	0.66	0.20, 2.20	0.5001
Healthcare empowerment (ref: low)	​	​	​	​	​	​	​	​	​	​	​	​	​	​	​	​	​	​
High	0.90	0.44, 1.81	0.7576	1.03	0.58, 1.84	0.9113	​	​	​	​	​	​	​	​	​	​	​	​
Healthcare avoidance (ref: no)																		
Yes	1.61	0.88, 2.94	0.1232	2.08	1.26, 3.46	**0.0047**	1.80	0.88, 3.67	0.1079	2.13	1.15, 3.94	**0.0169**	1.96	0.86, 4.47	0.1112	2.40	1.07, 5.42	**0.0349**
Physical health (ref: good)																		
Poor	0.46	0.23, 0.95	**0.0374**	0.74	0.43, 1.28	0.2841	0.51	0.22, 1.22	0.1316	0.65	0.32, 1.30	0.2210	0.51	0.20, 1.30	0.1593	0.73	0.30, 1.77	0.4720
Participation in medical research (ref: no)																		
Yes	0.53	0.29, 0.95	**0.0338**	0.55	0.34, 0.91	**0.0200**	0.88	0.44, 1.76	0.7208	0.90	0.49, 1.63	0.7225	1.12	0.49, 2.56	0.7810	1.35	0.60, 3.02	0.4720
Participation in genomic research (ref: no)																		
Yes	1.27	0.46, 3.49	0.6438	1.66	0.71, 3.85	0.2406	​	​	​	​	​	​	​	​	​	​	​	​
Beliefs on origins of gender identity	1.53	1.12, 2.08	**0.0077**	3.10	2.32, 4.15	**<0.0001**	​	​	​	​	​	​	0.88	0.57, 1.37	0.5659	1.32	0.85, 2.05	0.2124
Research team composition	1.31	1.00, 1.70	0.0502	1.56	1.25, 1.95	**0.0001**	​	​	​	​	​	​	1.31	0.89, 1.91	0.1721	1.42	0.96, 2.10	0.0768
Personal, community, and societal risks and benefits of TIGR	5.40	3.35, 8.76	**<0.0001**	14.68	8.87, 24.31	**<0.0001**	​	​	​	​	​	​	5.02	2.57, 9.80	**<0.0001**	14.48	7.30, 28.70	**<0.0001**

^a^ OR, odds ratio; aOR, adjusted odds ratio; 95% CI = 95% confidence interval. Bolded values indicate statistical significance at the *p* < 0.05 level.

In multivariable model 1, greater odds of being willing to participate in TIGR were associated with POC vs. White ethnoracial identity (aOR = 2.73, 95% CI = 1.35 to 5.53), food insecurity (aOR = 2.24, 95% CI = 1.16 to 4.33), and healthcare avoidance (aOR = 2.13, 95% CI = 1.15 to 3.94), while lower odds were associated with high vs. low medical mistrust (aOR = 0.26, 95% CI = 0.10 to 0.69). Uncertainty about participation was associated with POC ethnoracial identity (aOR = 2.50, 95% CI = 1.13 to 5.56) and lower vs. higher policy awareness (aOR = 2.56, 95% CI = 1.21 to 5.41), while lower odds of uncertainty were associated with high vs. low medical mistrust (aOR = 0.24, 95% CI = 0.08 to 0.68) and worse self-rated general health (aOR = 0.52, 95% CI = 0.27 to 0.99).

In multivariable model 2, after incorporating TIGR scale scores, ethnoracial identity, food insecurity, policy awareness, and medical mistrust were no longer statistically significantly associated with willingness to participate. Healthcare avoidance remained significantly associated with willingness (aOR = 2.40, 95% CI = 1.07 to 5.42). Only one factor from the TIGR scale, “Personal, Community, and Societal Risks and Benefits of TIGR,” remained significantly associated with willingness (aOR = 14.48, 95% CI = 7.30 to 28.70) and being unsure (aOR = 5.02, 95% CI = 2.57 to 9.80), meaning that positive perceptions of TIGR’s impact at the personal, communal, and societal levels were associated with higher odds of being willing or uncertain to participate rather than unwilling.

## Discussion

This nationwide survey of trans adults in the U.S. found high variability in perspectives on TIGR, supporting prior qualitative (Rajkovic et al., 2022; Hammack-Aviran et al., 2022) and quantitative (Theisen et al., 2024) studies reporting no clear consensus on the level of support for TIGR among trans communities. A large proportion of respondents were uncertain about the genetic origins of gender identity, perhaps reflecting the ambiguity surrounding TIGR (Rajkovic et al., 2022). Although some participants viewed TIGR as potentially beneficial because it could help individuals accept their own identity, lead to greater societal acceptance, or help fight discriminatory laws, the majority of participants were concerned about the potential harms that could result from TIGR, especially if a genetic explanation for trans identity were discovered. Many participants believed that such a finding would be used to harm the trans community. Still, most respondents would be willing to participate in TIGR, despite concern about the potential for harm, although those with higher TIGR scale scores—and therefore, more positive perceptions of TIGR—were significantly more willing to participate in TIGR or express uncertainty about participation. However, being unsure about participation could convey openness to TIGR on the part of some participants (*i.e.*, not a clear or definite unwillingness to participate).

Although research seeking a genetic source for LGBTQ+ identity is not new, behavioral genetics research has at times amplified and popularized this study, moving it beyond the laboratory and into public discourse (O’Riordan, 2012). The original “gay gene” study in 1993 (Hamer et al., 1993), although never replicated and met with criticism for both its study design and ethical implications, has become a highly visible cultural reference point (O’Riordan, 2012; Price, 2018). Its reception and reproduction in mainstream media have illustrated several dynamics that remain salient for TIGR: a readiness to apply biological essentialism to marginalized populations, echoing a harmful historical pattern; the pathologization of queer identities; and the tendency for complex findings to be simplified into deterministic narratives. Altogether, this has shaped public expectations about what genetics can reveal about identity, influencing how subsequent TIGR research may be understood or misused. Accordingly, our results should be interpreted against this cultural and social context, which may explain why many respondents anticipated harm even while expressing openness to participation.

Concern about the risks associated with the use of TIGR data or findings should not be surprising, given the history of biological essentialism present in behavioral genetics research, the pathologization of trans communities, and eugenic efforts to limit gender expression (Ordover, 2023; Stern, 2024). Most of our study population would feel more accepting (*i.e.*, more comfortable) with TIGR if the research team included members of the trans and LGBTQ+ communities, perhaps suggesting that the potential for harm might be addressed in some way by intentional composition of the research team. Although Theisen et al. (2024) reported the same finding, the proportion of participants who would feel more comfortable with LGBTQ+ research team members was higher in our sample. Direct (*i.e.*, the belief that TIGR would be used to harm trans communities) and indirect (*i.e.*, related to the issue of medical mistrust) harm was a consistent and significant concern.

Nearly three-quarters of our study population (73.9%) reported high medical mistrust, which was associated with less favorable views on TIGR and lower willingness to participate in TIGR (in the first multivariable model). This result is consistent with previous work that has found high medical mistrust among trans populations in the context of genomics (Angelo et al., 2022) and research that has found medical mistrust to be associated with lower participation in genomics research (O’Connor and Garrison, 2025). High medical mistrust may reflect concerns about exploitation, misuse of data, and lack of accountability in the context of prior harms experienced by trans people in healthcare settings (Hanssmann, 2023). It may also reflect a general mistrust of genetics among broader populations, given previous harms associated with genomics research (Rebbeck et al., 2022; Popejoy and Fullerton, 2016; Ordover, 2023). Mistrust is an issue of fundamental importance for research and healthcare settings. Our findings underscore the importance of acknowledging and considering the history of mistrust while developing strategies to build trust and transparency.

Perspectives on the risks and benefits of TIGR differed across social, health, and policy contexts. With regard to social contexts, individuals with minoritized ethnoracial identities had more favorable views of TIGR than their White counterparts, a finding that differs from prior work describing POC’s skepticism surrounding which populations benefit from genomics research (Ridgeway et al., 2019; Fisher et al., 2020; Scharff et al., 2010; Murphy et al., 2009). Alternatively, individuals in our study with self-reported disabilities had significantly less favorable views of TIGR (compared to those without disabilities), a finding that differs from a similar study that found high support for precision medicine research (although not for TIGR, specifically) among individuals with disabilities (Sabatello et al., 2019). Participants with disabilities in this sample may view TIGR more critically due to the historical and ongoing misuse of genetics in ableist frameworks that pathologize disability (Da Silva and Hubbard, 2024; Mintz et al., 2024; Madden et al., 2024). To ensure inclusivity in genomics research, care should be taken to engage trans people of color and those with disabilities.

Regarding the policy context, individuals residing in mixed *versus* negative policy environments had less favorable views of TIGR. Because we did not assess respondents’ reasons for their views, we cannot identify the mechanisms underlying this association. In light of the evolving policy landscape and increasing anti-trans legislation, future studies should directly measure policy-related concerns (*e.g.,* perceived legal risk and anticipated misuse of findings) to clarity how policy climate relates to TIGR attitudes and participation.

Finally, when TIGR perspectives were added to the multivariable model, the social, economic, health, and policy variables were no longer significantly associated with willingness to participate in TIGR, suggesting that views of TIGR may mediate associations between these factors and willingness and highlighting the importance of TIGR perspectives, particularly consideration of risks and benefits, in the engagement and participation of trans communities in research. We recommend that genetic researchers employ community-based frameworks (Lemke et al., 2022), in which they work closely with trans individuals to understand their political and social contexts and to explore how the evolving policy environment may affect perspectives on TIGR. Genomics researchers seeking to engage trans people should prioritize trust-building strategies alongside efforts to ensure participant safety and address risks, including the potential for harm, as these approaches may encourage future research participation.

Several limitations are important to consider in interpreting study findings. First, our sampling approach ensured that we recruited and enrolled a diverse sample, but the sample may not be representative or generalizable to the U.S. trans population. Second, we grouped ethnoracial identity into POC or White for analyses due to sample size; thus, we were unable to examine the unique perspectives of varied ethnoracial identity groups. Third, because the two-spirit measure was only displayed to respondents who selected American Indian or Alaska Native, we may have missed Indigenous respondents who identify as two-spirit but did not endorse this ethnoracial identity. Fourth, our sample may reflect selection bias in terms of political environment such that trans adults residing in more supportive policy environments may have been more likely to identify for recruitment and participate, potentially biasing estimates of differences in TIGR perspectives and willingness to participate across policy environments. Finally, our results on willingness to participate should be interpreted with caution; the percentage of those who would be willing to participate is likely high due to selection bias since participants who are already involved in research would be more willing to be involved in future research and because participants were being asked about willingness to engage in TIGR by researchers.

Although our study did not explore how perspectives on TIGR and willingness to participate in such research may translate behaviorally into actual future research participation, our findings point to key issues that should shape how TIGR is designed and communicated. First, it is essential to understand that how trans individuals interpret and assess TIGR’s aims and downstream research uses is central to research participation. At the same time, researchers must work closely with and build trust among trans individuals to understand their differing views on TIGR and distinct sociopolitical contexts while acknowledging past harms and potential future risks. To ensure that TIGR advances ethically and equitably, researchers should prioritize community engagement, transparency, and trust-building. They must address the specific concerns of those who are most skeptical of and least willing to participate in genomics research.

## Data Availability

The datasets presented in this article are not readily available. Data will be made available upon reasonable request. Access requires submission of a concept sheet for investigator review and completion of the necessary data use agreements between institutions. Requests to access the datasets should be directed to edusic@umich.edu.
