# Supplementary material for: Perceptions of and willingness to participate in genomics research among a U.S. sample
Source: Front Genet. 2026 Sep 9;17:1808576. doi: 10.3389/fgene.2026.1808576 (PMC13597166; doi:10.3389/fgene.2026.1808576)
Supplement: Supplementary file 1 [file Supplementaryfile1.docx]

Supplemental Material 3.  We collected ZIP codes to categorize geographic location by U.S. census region (Midwest, Northeast, Southeast, West) and by Gender Identity Policy Environment using a measure from the Movement Advancement Project (Negative: indicating states with anti-trans policy environments; Positive: indicating states with trans-friendly policy environments; and Mixed: indicating states with both negative and positive trans policies). Movement Advancement Project (MAP, link: <https://www.lgbtmap.org/)> is a nonprofit organization that collects data on LGBTQ+ and trans specific laws and policies in each state. The policy tally scores the laws and policies within each state that shape LGBTQ+ people’s lives, experiences and equality. The major categories of laws covered by the policy tally include: relationship & parental recognition, nondiscrimination, religious exemption, LGBTQ youth, healthcare, criminal justice, and identity documents. On MAP’s website, states are placed in five categories: high, medium, fair, low, and negative. In our analysis negative refers to states ranked as negative by MAP, positive refers to states ranked as high, and mixed refers to states ranked as medium/fair/low. In trans health research, the MAP is commonly used as a measure of trans-friendly policy environment.
