# Supplementary material for: Perceptions of and willingness to participate in genomics research among a U.S. sample
Source: Front Genet. 2026 Sep 9;17:1808576. doi: 10.3389/fgene.2026.1808576 (PMC13597166; doi:10.3389/fgene.2026.1808576)
Supplement: Supplementary file 2 [file Table1.docx]

| **Supplemental Table 1.** Exploratory Factor Analysis of Transgender Identity Genomics Research (TIGR) Scale | | | | |
| --- | --- | --- | --- | --- |
| **Item** | **Factor Loading** | **Cronbach's Alpha** | **Eigenvalue** | **Proportion of Variance Explained** |
| Overall TIGR Scale |  | 0.88 |  |  |
| **Beliefs on Origins of Gender Identity** |  | 0.74 | 1.74 | 0.18 |
| There is a genetic explanation for transgender identity. | 0.79 |  |  |  |
| I believe that identifying as LGBTQ+ (Lesbian, Gay, Bisexual, Transgender, Queer, +) runs in families. | 0.58 |  |  |  |
| Transgender identity occurs during development, before the baby is born. | 0.63 |  |  |  |
| **Research Team Composition** |  | 0.78 | 1.35 | 0.27 |
| I would feel more comfortable if the team conducting genetic research on transgender identity included members of the LGBTQ+ community. | 0.76 |  |  |  |
| I would feel more comfortable if the team conducting genetic research on transgender identity included people who identified as transgender. | 0.84 |  |  |  |
| **Personal, Community, and Societal Risks and Benefits of TGIR** |  | 0.89 | 5.78 | 0.55 |
| If there was a genetic reason that some people were transgender, I would want to know about it. | 0.62 |  |  |  |
| The idea of searching for a genetic reason for transgender identity makes me nervous. | 0.83 |  |  |  |
| The idea of searching for a genetic reason for transgender identity makes me hopeful. | 0.64 |  |  |  |
| Knowing more about a genetic reason for transgender identity would help me to better accept my own transgender identity. | 0.52 |  |  |  |
| If a genetic reason for transgender identity were discovered, it would lead to significant benefits for the transgender community. | 0.78 |  |  |  |
| If a genetic reason for transgender identity were discovered, it would be used to harm the transgender community. | 0.73 |  |  |  |
| Understanding the genetic reason for transgender identity would lead to better acceptance of transgender individuals by the non-transgender community. | 0.57 |  |  |  |
| If a genetic reason for transgender identity exists, understanding that reason would help to fight against discriminatory laws such as the "bathroom bills". | 0.65 |  |  |  |
| When I think about research that might find a genetic reason for transgender identity, I think the risks outweigh the benefits overall. | 0.44 |  |  |  |
