# Supplementary material for: Perceptions of and willingness to participate in genomics research among a U.S. sample
Source: Front Genet. 2026 Sep 9;17:1808576. doi: 10.3389/fgene.2026.1808576 (PMC13597166; doi:10.3389/fgene.2026.1808576)
Supplement: Supplementary file 3 [file Table2.docx]

| **Supplemental Table 2.** Correlations of Individual Items in the Transgender Identity Genomics Research (TIGR) Scale. | | | | | | | | | | | | | | |
| --- | --- | --- | --- | --- | --- | --- | --- | --- | --- | --- | --- | --- | --- | --- |
|  | **TIGR1** | **TIGR2** | **TIGR3** | **TIGR4** | **TIGR5^a^** | **TIGR6** | **TIGR7** | **TIGR8** | **TIGR9^a^** | **TIGR10** | **TIGR11** | **TIGR12** | **TIGR13** | **TIGR14^a^** |
| **TIGR1** | 1 |  |  |  |  |  |  |  |  |  |  |  |  |  |
| **TIGR2** | 0.53^***^ | 1 |  |  |  |  |  |  |  |  |  |  |  |  |
| **TIGR3** | 0.57^***^ | 0.37^***^ | 1 |  |  |  |  |  |  |  |  |  |  |  |
| **TIGR4** | 0.43^***^ | 0.19^***^ | 0.33^***^ | 1 |  |  |  |  |  |  |  |  |  |  |
| **TIGR5^a^** | 0.25^***^ | 0.14^**^ | 0.14^**^ | 0.47^***^ | 1 |  |  |  |  |  |  |  |  |  |
| **TIGR6** | 0.60^***^ | 0.34^***^ | 0.44^***^ | 0.62^***^ | 0.48^***^ | 1 |  |  |  |  |  |  |  |  |
| **TIGR7** | 0.48^***^ | 0.29^***^ | 0.34^***^ | 0.45^***^ | 0.38^***^ | 0.65^***^ | 1 |  |  |  |  |  |  |  |
| **TIGR8** | 0.55^***^ | 0.29^***^ | 0.38^***^ | 0.58^***^ | 0.52^***^ | 0.73^***^ | 0.59^***^ | 1 |  |  |  |  |  |  |
| **TIGR9^a^** | 0.35^***^ | 0.25^***^ | 0.18^***^ | 0.39^***^ | 0.58^***^ | 0.46^***^ | 0.39^***^ | 0.55^***^ | 1 |  |  |  |  |  |
| **TIGR10** | 0.48^***^ | 0.29^***^ | 0.40^***^ | 0.46^***^ | 0.34^***^ | 0.63^***^ | 0.52^***^ | 0.71^***^ | 0.43^***^ | 1 |  |  |  |  |
| **TIGR11** | 0.51^***^ | 0.27^***^ | 0.36^***^ | 0.50^***^ | 0.42^***^ | 0.63^***^ | 0.54^***^ | 0.73^***^ | 0.45^***^ | 0.70^***^ | 1 |  |  |  |
| **TIGR12** | 0.11^*^ | -0.01 | 0.11^*^ | 0.21^***^ | 0.05 | 0.17^***^ | 0.11^*^ | 0.12^**^ | -0.02 | 0.14^**^ | 0.21^***^ | 1 |  |  |
| **TIGR13** | -0.00 | -0.14^**^ | 0.06 | 0.08 | -0.12^**^ | -0.02 | -0.04 | -0.07 | -0.16^***^ | -0.02 | -0.00 | 0.63^***^ | 1 |  |
| **TIGR14^a^** | 0.16^***^ | 0.10^*^ | 0.15^**^ | 0.24^***^ | 0.33^***^ | 0.27^***^ | 0.19^***^ | 0.28^***^ | 0.32^***^ | 0.27^***^ | 0.27^***^ | 0.03 | -0.00 | 1 |
| **Supplemental Table 2.** Correlations of Individual Items in the Transgender Identity Genomics Research (TIGR) Scale.  TIGR1: There is a genetic explanation for transgender identity.  TIGR2: I believe that identifying as LGBTQ+ (Lesbian, Gay, Bisexual, Transgender, Queer, +) runs in families.  TIGR3: Transgender identity occurs during development, before the baby is born.  TIGR4: If there was a genetic reason that some people were transgender, I would want to know about it.  TIGR5: The idea of searching for a genetic reason for transgender identity makes me nervous.  TIGR6: The idea of searching for a genetic reason for transgender identity makes me hopeful.  TIGR7: Knowing more about a genetic reason for transgender identity would help me to better accept my own transgender identity.  TIGR8: If a genetic reason for transgender identity were discovered, it would lead to significant benefits for the transgender community.  TIGR9: If a genetic reason for transgender identity were discovered, it would be used to harm the transgender community.  TIGR10: Understanding the genetic reason for transgender identity would lead to better acceptance of transgender individuals by the non-transgender community.  TIGR11: If a genetic reason for transgender identity exists, understanding that reason would help to fight against discriminatory laws such as the "bathroom bills".  TIGR12: I would feel more comfortable if the team conducting genetic research on transgender identity included members of the LGBTQ+ community.  TIGR13: I would feel more comfortable if the team conducting genetic research on transgender identity included people who identified as transgender.  TIGR14: When I think about research that might find a genetic reason for transgender identity, I think the risks outweigh the benefits overall.  ^a^Items were reverse scored.  ^*^p<0.05  ^**^p<0.01  ^***^p<0.001 | | | | | | | | | | | | | | |
